# Why do humans undergo an adiposity rebound? Exploring links with the energetic costs of brain development in childhood using MRI-based 4D measures of total cerebral blood flow

**DOI:** 10.1038/s41366-022-01065-8

**Published:** 2022-02-08

**Authors:** Jacob E. Aronoff, Ann Ragin, Can Wu, Michael Markl, Susanne Schnell, Ali Shaibani, Clancy Blair, Christopher W. Kuzawa

**Affiliations:** 1 Department of Anthropology, Northwestern University, Evanston, IL USA; 2 Department of Radiology, Northwestern University Feinberg School of Medicine, Chicago, IL USA; 3 Department of Medical Physics, Memorial Sloan Kettering Cancer Center, New York, NY USA; 4 Department of Biomedical Engineering, Northwestern University McCormick School of Engineering, Chicago, IL USA; 5 Department of Medical Physics, Institute of Physics, University of Greifswald, Greifswald, Germany; 6 Department of Neurosurgery, Northwestern University Feinberg School of Medicine, Chicago, IL USA; 7 Department of Population Health, NYU School of Medicine, New York, NY USA; 8 Department of Applied Psychology, NYU Steinhardt, New York, NY USA; 9 Institute for Policy Research, Northwestern University, Evanston, IL USA

**Keywords:** Metabolism, Risk factors

## Abstract

**Background:**

Individuals typically show a childhood nadir in adiposity termed the adiposity rebound (AR). The AR serves as an early predictor of obesity risk, with early rebounders often at increased risk; however, it is unclear why this phenomenon occurs, which could impede understandings of weight gain trajectories. The brain’s energy requirements account for a lifetime peak of 66% of the body’s resting metabolic expenditure during childhood, around the age of the AR, and relates inversely to weight gain, pointing to a potential energy trade-off between brain development and adiposity. However, no study has compared developmental trajectories of brain metabolism and adiposity in the same individuals, which would allow a preliminary test of a brain-AR link.

**Methods:**

We used cubic splines and generalized additive models to compare age trajectories of previously collected MRI-based 4D flow measures of total cerebral blood flow (TCBF), a proxy for cerebral energy use, to the body mass index (BMI) in a cross-sectional sample of 82 healthy individuals (0–60 years). We restricted our AR analysis to pre-pubertal individuals (0–12 years, *n* = 42), predicting that peak TCBF would occur slightly after the BMI nadir, consistent with evidence that lowest BMI typically precedes the nadir in adiposity.

**Results:**

TCBF and the BMI showed inverse trajectories throughout childhood, while the estimated age at peak TCBF (5.6 years) was close but slightly later than the estimated age of the BMI nadir (4.9 years).

**Conclusions:**

The timing of peak TCBF in this sample points to a likely concordance between peak brain energetics and the nadir in adiposity. Inverse age trajectories between TCBF and BMI support the hypothesis that brain metabolism is a potentially important influence on early life adiposity. These findings also suggest that experiences influencing the pattern of childhood brain energy use could be important predictors of body composition trajectories.

## Introduction

In humans, fat stores typically decrease from infancy to childhood, reach a nadir in middle childhood, and then rebound as adiposity begins to increase from adolescence into adulthood. The age at which this “adiposity rebound” (AR) is experienced determines when individuals start to regain adiposity and is an important predictor of long-term trajectories of body composition and risk for overweight, obesity, and related metabolic diseases [1–4]. The age at rebound varies widely from 3 to 8 years [1, 5], with a study finding that a one year change in AR timing was associated with a 2.5 kg/m^2^ difference in the body mass index (BMI) in young adulthood [6]. These findings point to the AR, and factors that influence its timing, as an important predictor of population variation in risk for obesity.

One question that has received little attention, but that could provide important insights into variation in adiposity across populations, is why an AR occurs during childhood. It is generally assumed that changes in body composition during infancy and childhood trace to shifts in energy balance related to changes in intake and expenditure. For instance, it has often been noted that the tendency for adiposity to decline after infancy might relate to increases in physical activity during childhood [7, 8]. However, another potentially important influence on patterns of energy expenditure at this age, and which could shed light on the AR, was recently inspired by work documenting age-related changes in brain energy requirements [9, 10]. In humans, the cerebral metabolic rate of glucose (CMRglu), the principal substrate used by the brain, rises rapidly during the first few years of life and peaks during childhood [11] when the brain consumes twice the quantity of glucose of the adult brain [10, 11]. These changing energetic costs trace to developmental shifts in brain size, but more importantly, to neuronal processes related to cognitive development. Human brain development involves overproduction of energetically demanding synaptic connections, which peak in density in childhood and is followed by experience-dependent pruning and a corresponding reduction in energy expenditure [12–14]. At the brain’s lifetime peak energy expenditure at 4–5 years of age, its rate of glucose consumption accounts for an estimated 66% of the body’s resting energy expenditure, or roughly three times the fraction of the body’s metabolism devoted to the brain in adulthood (20–25%) [10].

The high energy demands of the brain have been implicated in the slowed growth observed during childhood [15]. Specifically, average growth velocities across childhood vary in a tight linear, inverse relationship with estimated average CMRglu: when brain expenditure is rising during infancy and childhood, the rate of body weight gain declines in parallel [10]. The childhood peak in brain expenditure coincides with the slowest rate of weight gain in the lifecycle, before weight gain begins to increase again at ages when synaptic pruning diminishes cerebral metabolic rate [10]. In these analyses, weight velocities and the percentage of resting metabolic rate (% RMR) to the brain were linearly, inversely related from infancy until approximately early adolescence (age 14), supporting the hypothesis that the pattern of human growth evolved to balance the shifting strength of energetic trade-offs with brain development. A subsequent study replicated this finding although the age range of the sample was narrower: in this second sample, declining % RMR devoted to the brain in late childhood was linearly and inversely related to the increasing rate of weight gain [16].

In addition to observed associations between brain energy requirements and weight gain, converging evidence points to a more generalized trade-off between energy expenditure on the brain and adipose tissue across the lifecycle [9]. In both children and adults, inverse associations have been found between measures of adiposity such as BMI, and the volume or thickness of cortical and subcortical structures throughout the brain [17–20]. Similarly, work identifying the genetic architecture of obesity points to strongest and most consistent links between adiposity and brain- and neuronally-expressed genes, some of which influence energetically demanding processes like synaptic function, long-term potentiation and neurotransmitter signaling [21–23].

Based upon these various lines of evidence, it has been proposed that the developmental nadir in adiposity may represent an outcome of peak energy requirements of brain development [9]. If this hypothesis is correct, factors that influence the timing or magnitude of brain energy use, whether environmental factors that shift the pace of cognitive development, or educational or other features of the rearing environment that influence peak brain expenditure, could influence individual and population variation in the trajectory of body composition development [9]. As a first step in exploring this hypothesis, here we compare age trajectories of the BMI and total cerebral blood flow (TCBF), quantified in vivo using advanced, non-invasive 4D-flow magnetic resonance imaging (MRI), in a sample of 82 healthy participants ages 0–60 years. We report the relationship between BMI and TCBF across the full age range for reference purposes. However, since the AR is a childhood phenomenon, we focus our AR analysis specifically on the pre-pubertal members of the sample (0–12 years, *n* = 42). Previous studies have shown that local glucose metabolism is closely coupled to cerebral blood flow [24, 25]. Moreover, developmental changes in TCBF show close parallels to the age-related trajectory in CMRglu [16, 26, 27], indicating that TCBF can serve as a non-invasive proxy for CMRglu. We hypothesized [1] that developmental changes in average TCBF will inversely track changes in average BMI in this sample and [2] that age at peak TCBF will closely approximate the age at AR. We hypothesized that the BMI nadir would slightly precede the TCBF peak, based on previous studies reporting that the developmental nadir in the BMI occurs earlier than the nadir in directly-measured adiposity, ranging from several months to a few years prior [28–30]. This reflects the fact that the BMI captures both lean and fat tissue and that, unlike fat stores, lean mass is not lost at the nadir but continues to increase in parallel with linear growth.

## Subjects and methods

The data used here include measures of TCBF and BMI (kg/m^2^) obtained from 82 Chicago area residents ranging in age from 0 to 60 years and acquired as part of a larger investigation of blood flow changes with age, as detailed elsewhere [27]. Briefly, individuals were screened for cardio- and cerebrovascular problems, ECG and high blood pressure (>160/90 mm Hg). Data were collected under IRB approval and in accordance with the Health Insurance Portability and Accountability Act guidelines. Parental consent was obtained for individuals ages 0–11 years, while adolescent assent was obtained for participants aged 12–17 years in addition to parental consent. Children ages 0–5 years were anesthetized or sedated using inhalational anesthetics (sevoflurane, Ultane; Abbott Laboratories, Inc). Flow imaging was successfully performed in all participants on clinical MRI scanners (Magnetom 1.5 T Aera or 3 T Skyra, Siemens Healthineers, Erlangen, Germany). 4D flow data preprocessing to filter background noise and correct for velocity aliasing and phase offset errors was done using custom Matlab tools (MathWorks, Natick, MA). Imaging data were imported into commercial software (EnSight; CEI, Apex, NC) for individual vascular flow quantification, using the 3D phase-contrast MR angiogram (PC-MRA, calculated from 4D flow MRI based on absolute velocities weighted by magnitude data) for anatomic orientation, as detailed elsewhere [27, 31]. The PC-MRA was used to mask velocity vectors allowing blood flow visualization and quantification within vessel walls. 2D analysis planes were manually positioned at predefined anatomic landmarks for internal carotid arteries (ICAs; straight section between lacerum C3 and cavernous C4 segments) and the basilar artery (BA; middle portion between anterior inferior cerebellar artery and superior cerebellar artery). Mean blood flow over the cardiac cycle was automatically calculated for each plane. TCBF was quantified as cumulative flow measured over the cardiac cycle in left and right ICA’s and BA [32, 33].

### Statistical analysis

Generalized additive models (GAM) were used to describe and model age-related changes in TCBF and BMI as well as to provide predicted ages for the AR and peak TCBF in the pre-pubertal subsample (ages 0–12, *n* = 42). GAM allowed implementation of penalized cubic regression splines to model non-linear trends in both measures. In addition, GAM allowed a test of whether individuals with higher TCBF for their age also had lower BMI (statistical significance determined at *p* < 0.05). A TCBF-to-CMRglu conversion was used based on a previous study [34], allowing comparison of our data to direct CMRglu measurements published by Kuzawa et al. (2014). The analysis was conducted in R (version 4.0), while the mgcv package [35] was used to fit GAM.

## Results

**Fig. 1:**
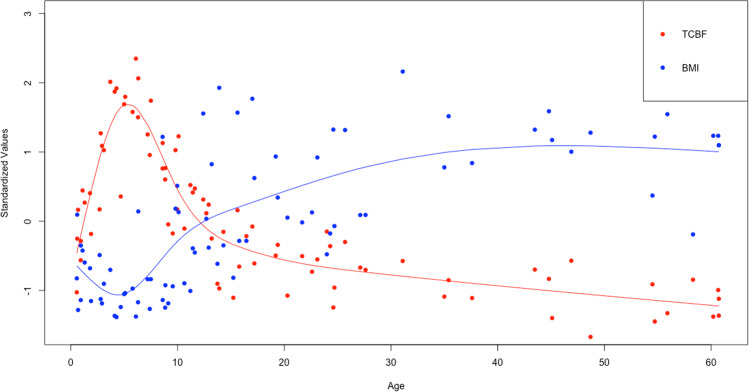
Age trajectories of BMI and TCBF (full sample, *n* = 82, ages 0–60 years) for reference. Lines are predicted means from fitted GAM. Values were standardized (mean = 0, SD = 1) to provide unitless comparison. Note: analysis sample consisted only of the 42 individuals ages 0–12 years.

**Fig. 2:**
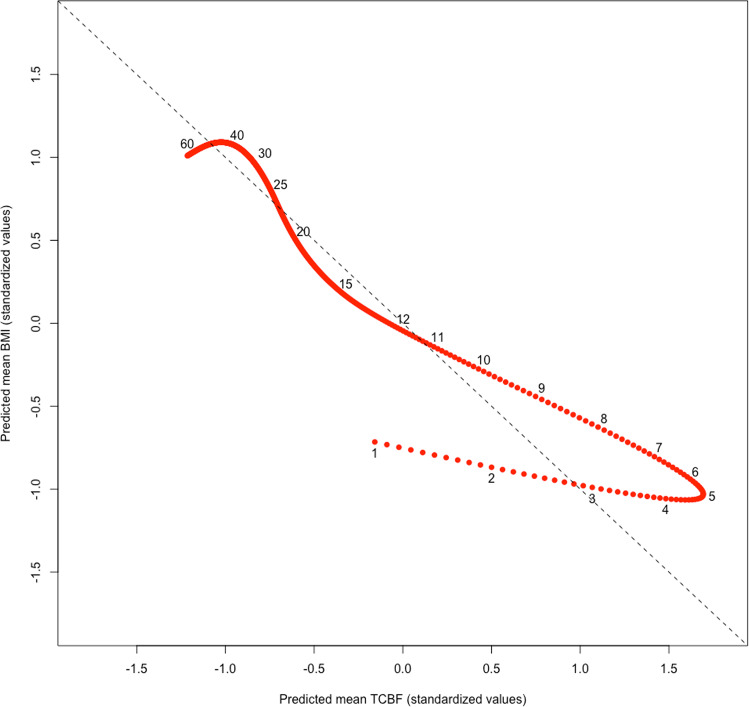
Association between standardized (mean = 0, SD = 1) TCBF and BMI predicted means across ages 0–60 years (full sample, *n* = 82) for reference. Predicted means were obtained from GAM shown in Fig. 1. Dotted line represents a slope of −1.

For reference, Fig. 1 plots BMI and TCBF across the full age range of the sample (*n* = 82), illustrating the general tendency for changes in BMI and TCBF to relate inversely across the lifecycle. Figure 2 visualizes the relationship between TCBF and BMI across ages using GAM-predicted standardized mean values (mean = 0, SD = 1), again including adults for reference. TCBF increases sharply until the peak around age 5 years. Although BMI changes inversely track with these changes in early life, they are relatively modest as reflected in the shallow slope. Beginning in adolescence and persisting into adulthood, changes in BMI continue to inversely track changes in TCBF but with proportionate changes, indicated by the data points falling largely on the dotted line representing a slope of −1.

Among the 42 children (0–12 years of age) selected for the analysis of the AR, there were 25 girls and 17 boys (Table 1). GAM-predicted mean values for TCBF and BMI across ages indicated an age of 5.6 years for peak TCBF and a BMI nadir age of 4.9 years (Table 2), close to the peak CMRglu age of 5.2 years reported by Kuzawa et al. (2014). We also include age trajectories of brain volume and ascending aortic output, reflecting total cardiac output, for reference in Supplementary Table S1. Both show steady increases with age, including while TCBF is declining following the peak at 5.6 years. Using the TCBF-to-CMRglu conversion derived from Settergren et al. (1980), estimated peak CMRglu for our sample was 143.2 g/day, very close to the PET-based peak estimate of 146 g/day for girls from Kuzawa et al. (2014), but lower than the 167 g/day for boys. After accounting for the effect of age using a GAM shown in Table 3, TCBF and BMI were not associated (*p* ~ 0.8), indicating that having a higher or lower TCBF than predicted for one’s age did not predict corresponding differences in the BMI in this sample.

**Table 1:** Descriptive statistics for the analysis sample spanning infancy-12 years (*n* = 42).

	Mean ± SD	Range
Age (years)	6.2 ± 3.9	(0, 12)
TCBF (ml/s)	19.5 ± 3.9	(10.9, 27.3)
BMI (kg/m^2^)	18.1 ± 3.6	(14.3, 30.0)
% Female	60%

**Table 2:** Predicted mean values for BMI nadir, peak TCBF, and estimated peak CMRglu^a^ for the analysis sample spanning infancy-12 years (*n* = 42).

	AR	TCBF peak	CMRglu peak (estimated^b^)
Age (years)	4.9	5.6	5.6
BMI (kg/m^2^)	16.6		
TCBF (ml/s)		24.1	
CMRglu (g/day)			143.2

^a^Predicted mean values were obtained from fitting GAM. Values at yearly intervals are reported in Supplemental Table S1.

^b^CMRglu was estimated from TCBF using a conversion obtained from Settergren, Lindblad, & Persson, 1980.

**Table 3:** GAM predicting BMI after adjusting for non-linear age effect (*n* = 42) for the analysis sample spanning infancy-12 years (*n* = 42).

	*β* (SE)	*P* value
Female	−0.75 (1.03)	0.47
TCBF	0.05 (0.18)	0.78

## Discussion

While much attention has been paid to the widely observed phenomenon of the AR, the question of why the AR occurs during childhood has been understudied. Clarifying the determinants of the AR could help facilitate a deeper understanding of lifecourse influences on body composition trajectories and related disease risk. Here we present evidence that TCBF peaks at around the same age as the AR, as indexed using the BMI. The age of peak TCBF in our sample, 5.6 years, is close to the age of peak CMRglu reported elsewhere using PET and MRI data (Kuzawa et al. 2014). We also find that age trajectories of TCBF and BMI in the sample are inversely correlated, suggesting brain energy requirements might be linked developmentally with changes in adiposity [9].

This finding has implications for understanding risk for overweight/obesity and metabolic disease and is consistent with converging evidence linking brain metabolism and adiposity. In both children and adults, negative associations have been reported between certain cognitive functions like executive function and adiposity [36]. Similarly, adiposity has been found to be inversely associated with frontal gray matter volumes as well as global gray matter blood flow [17, 37–39]. While most studies are cross-sectional, emerging longitudinal evidence also supports an association between the energetics of brain development and body composition trajectories. For example, the rate of decline in BMI preceding the AR has been associated with greater gain in executive function, which is largely orchestrated by the prefrontal cortex, a region with particularly high energy demands during childhood [9, 40]. Taken together, these findings point to brain metabolism as a potentially promising research area for clarifying developmental processes that contribute to body weight and composition trajectories.

There are likely multiple pathways regulating both brain energy consumption and adiposity during childhood that could be the target of future studies. For example, the brain has direct control over physiological mechanisms regulating its own metabolism as well as adiposity stores, including insulin production and stimulation of lipolysis [41, 42]. This relationship is also potentially bidirectional. For instance, as adiposity increases, adipocytes and resident macrophages secrete pro-inflammatory cytokines, such as interleukin-6, that have been associated with reduced brain volumes and cognitive function [43–45], potentially diminishing energy expenditure in parallel. Adipose-tissue secretion of adiponectin, which has anti-inflammatory effects in the brain, also decreases with increasing adiposity, potentially reinforcing a pro-inflammatory state [46, 47]. Secretion of leptin, which promotes brain growth and development, increases with greater adiposity, although this eventually leads to leptin-resistance that is thought to impair brain function [47–49]. Finally, genetic pleiotropy is another likely mechanism. Previous studies have identified genes involved in both brain function and adiposity development [21–23], while a meta-analysis identified BMI-associated gene variants that have co-expression patterns associated with synaptic maintenance and function, a major component of the brain’s metabolic requirements [50].

The apparent concordance between the developmental trajectories of the brain and adiposity also highlights the brain as a potential pathway linking early life experiences with obesity risk. Recent work has shown that total energy expenditure during childhood is similar across populations varying widely in patterns of physical activity and environmental exposures [51], suggesting that trade-offs operate between the body’s various functions to maintain total expenditure within a constrained range. In light of this, it is of interest that measures reflecting the magnitude of investment in brain structures, including cortical thickness, gray matter volume and even brain volume, are increased among children in higher socioeconomic and enriched rearing environments [52, 53], while conversely cerebral blood flow is decreased in the context of low SES or traumatic experiences [54]. We speculate that factors influencing early cognitive stimulation, or stressors that impair cognitive development, could have long-term impacts on the fraction of the body’s total expenditure devoted to the brain, with possible impacts on competing expenditures like growth and fat deposition [9]. Early adversity and stressors could similarly alter body composition trajectories by accelerating brain maturation [54], possibly leading to an earlier peak in brain metabolism and resulting in an earlier AR and greater adiposity.

Our study also has implications for the impact of early nutritional adversity on brain development in lower resource settings, where undernutrition may be more prevalent and severe. The AR generally occurs at later ages in children from lower income and undernourished populations [5, 55]. This raises the question of whether the age of peak brain energy needs is also delayed, matching the AR, with possible implications for cognitive development. Numerous studies have found relationships between undernutrition in childhood and cognitive deficits [56–58], which might be reflective of a later peak in energetically costly developmental processes. Testing this possibility will require obtaining measures of brain metabolism, or reliable proxies, across infancy and childhood in populations varying in nutritional status and timing of the AR. Although obtaining 4D-flow TCBF requires MRI facilities and operator training that may be prohibitive in some research settings, other options, such as ultrasound [59–61], near infrared spectroscopy (NIRS) [62] and portable low-field MRI units [63] provide opportunities to test these hypotheses. There are important trade-offs that must be considered with each method. For instance, it has been argued that the childhood peak in CMRO_2_ could be blunted by as much as 30% compared to CMRglu [26], which, if correct, would obscure the apparent strength of the relationship between brain metabolism and adiposity if changes in cerebral oxygen metabolism are assessed. Similarly, studies that report age trajectories in TCBF using ultrasound show a markedly attenuated childhood increase in TCBF relative to adult values, compared to the 4D-flow based measures reported here [59]. These and other measurement challenges underscore that nuance will be required in testing for relationships between brain metabolism and body composition change.

There are important limitations to this study. Most notably, these data are cross-sectional, which does not allow us to observe changes in the BMI or TCBF within individuals over time. We also did not have direct measures of CMRglu, which are necessary to quantify energy substrate use by the brain. However, cerebral blood flow is closely coupled to glucose metabolism, while TCBF and CMRglu follow similar, parallel age trajectories in humans [16, 24–27]. Further, the exposure to radiation involved in direct CMRglu measurement using PET presents ethical concerns that will likely constrain most studies of brain metabolism to blood flow estimates. We also relied on the BMI, which is a reflection not only of adiposity but also of lean mass. This could help explain why we did not see proportional declines in BMI matching increases in TCBF leading up to the AR (Fig. 2), as lean mass increases while fat mass decreases at this age [29].

Our reliance on the BMI also likely explains why the AR age was slightly earlier than peak TCBF, since previous studies have shown AR age estimates can be 0.4–2.9 years earlier when using the BMI compared to direct adiposity measures due to developmental increases in lean mass throughout childhood [28–30]. However, there could be other explanations for the lack of precise developmental concordance between the BMI nadir and peak TCBF in our sample. Although it is theoretically possible that reduced expenditure on physical activity could offset the brain’s energy needs and free up energy to store as adipose tissue, levels of physical activity increase from infancy through adolescence [7, 64] suggesting that this is an unlikely explanation. While we do not have data on immune function expenditure, lymphocyte numbers decline from infancy to adulthood [65], possibly allowing declining immune function expenditure to partially offset the brain’s needs. Finally, we do not have data on age-related changes in metabolism of other metabolically costly organs, which could also be a contributing factor.

Based on a collection of previous studies reporting inverse associations between adiposity and both cognitive function and frontal gray matter volumes [17, 36–38, 40], we predicted that individuals with lower TCBF for their age would also have relatively higher BMIs. Our small sample was likely underpowered to test this association, and the confounding influence of lean mass on the BMI could have also contributed to our null findings, as individuals with greater lean mass for their age could have both greater BMI and TCBF. However, brain volume was not associated with BMI after adjusting for age (*p* > 0.80; analysis not shown), arguing against this interpretation. Our null results testing a TCBF-BMI association at the individual level might suggest the relationship is more easily detectable at the aggregate, population level, owing to the greater reliability in mean changes, but with individual-level differences more challenging to detect.

In conclusion, we find that blood flow to the brain and the BMI follow inverse developmental trajectories during infancy and childhood, with peak TCBF occurring around the same age as the AR. These findings represent the first empirical test, and support, for the hypothesis that the AR is linked developmentally to the high energetic requirements of brain development at this age. These findings also raise the question of whether population differences in the timing of the AR are associated with corresponding variation in the time course of peak brain energetics and underlying developmental processes of cognitive development. The relationship between brain metabolism and adiposity during childhood points to the need for longitudinal studies with direct adiposity measures while also considering factors that could moderate or alter the timing or magnitude of brain cognitive development, including factors like environmental adversity, childhood nutritional sufficiency, or early educational enrichment programs.

## Supplementary information

Supplementary Materials

## Data Availability

The analysis code is available at: https://github.com/jakearonoff/blood-flow-bmi.
