# Supplementary material for: Why do humans undergo an adiposity rebound? Exploring links with the energetic costs of brain development in childhood using MRI-based 4D measures of total cerebral blood flow
Source: Int J Obes (Lond). 2022 Feb 8;46(5):1044–50. doi: 10.1038/s41366-022-01065-8 (PMC9050592; doi:10.1038/s41366-022-01065-8)
Supplement: Supplementary file 1 — Supplementary Materials [file 41366_2022_1065_MOESM1_ESM.docx]

**Table S1.** GAM predicted means for relevant variables

| Age (Y) | Weight (kg) | Height (m) | BMI (kg/m2) | TCBF (mL/s) | Brain Volume (mL) | Brain Perfusion (mL/min/100 mL) | Ascending Aortic output (AAo, mL/s) | TCBF/Aao |
| --- | --- | --- | --- | --- | --- | --- | --- | --- |
| 1 | 9.6 | 0.7 | 17.6 | 15.3 | 827.3 | 111.1 | 34.3 | 0.5 |
| 2 | 11.6 | 0.8 | 17.2 | 18.2 | 890.1 | 122.7 | 39.3 | 0.5 |
| 3 | 13.7 | 0.9 | 16.9 | 20.8 | 946.3 | 131.7 | 44.3 | 0.4 |
| 4 | 16.1 | 1 | 16.6 | 22.9 | 1008.2 | 136.5 | 49.3 | 0.4 |
| 5 | 18.9 | 1.1 | 16.6 | 23.9 | 1072.1 | 136.2 | 54.2 | 0.4 |
| 6 | 22.2 | 1.2 | 16.7 | 23.8 | 1131 | 130 | 59.2 | 0.4 |
| 7 | 26.1 | 1.2 | 17.1 | 22.8 | 1180.2 | 118 | 64.2 | 0.4 |
| 8 | 30.6 | 1.3 | 17.6 | 21.3 | 1218.2 | 103.3 | 69.2 | 0.3 |
| 9 | 35.5 | 1.4 | 18.4 | 19.7 | 1257.3 | 90.7 | 74.1 | 0.3 |
| 10 | 40.7 | 1.4 | 19.4 | 18.3 | 1297.4 | 84 | 79.1 | 0.3 |
| 11 | 46.3 | 1.5 | 20.4 | 17.5 | 1296.6 | 82.9 | 84.1 | 0.2 |
| 12 | 52 | 1.6 | 21.5 | 17.1 | 1230.6 | 85.4 | 89.1 | 0.2 |
